# Plasma miR-98-5p as a candidate biomarker of pain severity in oxaliplatin-induced peripheral neuropathy related to NGF and catecholamine dysregulation

**DOI:** 10.3389/fmolb.2026.1884789

**Published:** 2026-09-08

**Authors:** Yeongdon Ju, Go-Eun Choi, Aelee Jang

**Affiliations:** 1 Gimcheon University, Gimcheon-si, Republic of Korea; 2 Department of Biomedical Laboratory Science, College of Health Sciences, Catholic University of Pusan, Busan, Republic of Korea; 3 Next-Generation Industrial Field-Based Specialist Program for Molecular Diagnostics, Brain Busan 21 Plus Project, Graduate School, Catholic University of Pusan, Busan, Republic of Korea; 4 University of Ulsan, Ulsan, Republic of Korea

**Keywords:** cortisol, gastrointestinal cancer, microRNA, neuropathy, numeric rating scale, oxaliplatin

## Abstract

**Background:**

Oxaliplatin-induced peripheral neuropathy (OIPN) is a common and severe adverse event in patients with gastrointestinal (GI) cancer who undergo oxaliplatin-based chemotherapy. Assessments remain largely dependent on a numeric rating scale (NRS), which is needed for early detection and objective quantification of pain intensity.

**Methods:**

This study aimed to identify differentially expressed microRNAs (miRNAs) in plasma samples from 22 patients with GI cancer treated with oxaliplatin to elucidate the association between candidate miRNA expression levels, nerve growth factor (NGF), catecholamines, and NRS scores for peripheral neuropathic pain. High-throughput RNA sequencing was used to analyze miRNA expression profiles, and patients were stratified into Groups 1 (*n* = 10, no or mild pain) and 2 (*n* = 12, moderate or severe pain) based on their NRS scores. Correlations between variables were assessed using Spearman’s rank-order correlation (r = 0.4031, *P* = 0.0629).

**Results:**

In Group 2, hsa-miR-98-5p was significantly upregulated and demonstrated the greatest potential to stratify patients by pain severity among the candidate miRNAs (The area under the curve = 0.8083, 95% CI: 0.6222–0.9944). Gene ontology enrichment analysis revealed that hsa-miR-98-5p was significantly associated with critical neuronal injury and repair signaling pathways. Patients in Group 2 exhibited decreased norepinephrine and dopamine levels, elevated NGF levels, and elevated cortisol levels, indicating potential alterations in the OIPN neuromodulatory systems.

**Conclusion:**

These findings suggest that miR-98-5p is a candidate biomarker of pain severity in OIPN and may play a role in OIPN pathophysiology.

## 1 Introduction

Despite therapeutic advancements, gastrointestinal (GI) cancers, including gastric and colorectal cancers, remain a major global health burden (Zhang et al., 2023; Arnold et al., 2020; Deng et al., 2022). Oxaliplatin is a key chemotherapeutic agent for these malignancies but is frequently limited by neurotoxicity, a dose-limiting toxicity that impairs nerve signal transmission through both direct and indirect mechanisms (Rokhsareh et al., 2023; Cheng et al., 2023; Yildirim and Cengiz, 2020). Oxaliplatin-induced peripheral neuropathy (OIPN) significantly impairs quality of life and can necessitate treatment modification or discontinuation, highlighting the need for predictive biomarkers and mechanistic insights (van Haren et al., 2021; Gu et al., 2023; Kang et al., 2021; Miyamoto et al., 2022; Yang et al., 2021).

MicroRNAs (miRNAs) have emerged as promising candidates for biomarker discovery and therapeutic targeting in neuropathic conditions, including OIPN (Tavakoli Pirzaman et al., 2023). They regulate key molecular processes such as oxidative stress, inflammation, and neuronal apoptosis (Konovalova et al., 2019; Jiang et al., 2022; Zhao et al., 2023). However, their role in oxaliplatin-induced neurotoxicity remains unclear. Neuromodulators such as catecholamines (epinephrine, norepinephrine, and dopamine), nerve growth factor (NGF), and cortisol have been implicated in neuropathic pain pathophysiology through the modulation of nociceptor excitability, neurotransmission, and stress responses (Yang et al., 2025; Porojan et al., 2010; Tsigos et al., 1993; Sun and Wang, 2023). Although alterations in catecholamine and NGF levels have been studied in diabetic neuropathy and other pain models, their relationship with pain severity in OIPN has not been explored.

Pain in OIPN is most often quantified using the Numeric Rating Scale (NRS), a validated and widely used clinical tool (Nugent et al., 2021; Alghadir et al., 2018; Krebs et al., 2007). However, no prior studies have examined the association between miRNA expression and NRS-defined pain intensity in OIPN, nor have they integrated these findings with biochemical markers such as catecholamines, NGF, and cortisol.

In this study, we aimed to elucidate the potential of miRNAs as a candidate biomarker of pain severity in patients with gastrointestinal cancer receiving oxaliplatin by combining high-throughput miRNA profiling, bioinformatics, and biochemical analyses. We investigated the correlation between the differential expression of miRNAs and NRS pain scores. Furthermore, we evaluated the concurrent alterations in catecholamine, NGF, and cortisol levels to determine whether miRNA dysregulation represents a neuromodulatory pathway associated with OIPN severity.

## 2 Materials and methods

### 2.1 Study subjects

The present study utilized data derived from previously reported cohorts of patients with gastrointestinal cancer treated with oxaliplatin (Ju et al., 2022; Ju et al., 2024). The samples were prospectively collected at the time of the cohort enrollment; however, this analysis represents a secondary analysis addressing a distinct research question that was not examined in Ju et al., 2022 or Ju et al., 2024. Using an integrated cohort, we investigated the associations between plasma miRNA expression profiles, catecholamine, NGF levels, and neuropathic pain severity assessed by the NRS score. Of the 22 patients enrolled in the present study, 4 were derived from the previously reported oxaliplatin-treated gastric cancer cohort, and 12 were derived from the previously reported oxaliplatin-treated colorectal cancer cohort. The remaining 6 patients were newly enrolled and were not included in either prior publication (Supplementary Table S1). Importantly, although the patient-level cohorts partially overlap, the miRNA expression data analyzed in the present study were newly generated and therefore represent an independent profiling effort rather than a reanalysis of previously reported expression data. In this study, 22 patients with GI cancer were categorized into two groups according to pain severity. Ten patients had no or mild pain, and 12 patients had moderate to severe pain. Written informed consent was obtained from all participants prior to study enrollment and blood sample collection. The study was conducted in compliance with the Declaration of Helsinki and was approved by the Institutional Review Board of Pusan National University Hospital (approval number H-1803-014-065). This study was registered with the Clinical Research Information Service (registration number KCT0005353) to ensure transparency and reproducibility.

### 2.2 Pain screening NRS

Pain severity was evaluated following the completion of oxaliplatin-based chemotherapy at the Pusan National University Hospital. The research team collaborated with a gastroenterologist at our hospital. The principal investigator briefed the shift nurse in charge of the study, who screened and identified potential participants according to the inclusion criteria. The severity of OIPN symptoms was assessed using the NRS, a validated clinical tool for pain measurement (Jang et al., 2018). As part of the vital sign assessment, the nursing staff systematically evaluated the patients' pain by requesting that they rate the intensity of their current pain using an NRS ranging from 0 (no symptoms) to 10 (worst symptom imaginable). Pain intensity was stratified into mild, moderate, and severe categories according to the classification criteria established in a previous study (Bao et al., 2020). To evaluate the effects of the treatment on pain, the enrollment criteria mandated moderate-to-severe pain (Gewandter et al., 2017). The threshold for moderate pain was defined as a score of 4, which is consistent with the established standards commonly used in clinical practice and administrative reporting (Krebs et al., 2007).

### 2.3 RNA extraction from the plasma samples

Total RNA was isolated from plasma samples using TRIzol reagent (Invitrogen, Thermo Fisher Scientific, Waltham, MA, United States) according to the manufacturer’s guidelines. RNA concentration and purity were assessed using a NanoDrop 2000 spectrophotometer (Thermo Fisher Scientific). RNA quality was assessed by measuring the absorbance ratios at 260/280 and 260/230 nm to ensure the integrity and suitability of the samples for further analyses. After confirming RNA quality, transcriptomic profiling was conducted using RNA sequencing to quantify expression levels and investigate the transcriptome. Sequencing data provided a detailed overview of RNA expression patterns in the plasma samples A reliable framework for robust transcriptomic analysis was established by combining rigorous RNA extraction, quality assessment, and sequencing techniques.

### 2.4 Enzyme-linked immunosorbent assay

Plasma samples from patients with GI cancer who received oxaliplatin-based chemotherapy were analyzed for NGF, cortisol, and catecholamine levels using enzyme-linked immunosorbent assay (ELISA). NGF concentrations were determined using a human beta-NGF ELISA Kit (Cat. No. EHNGF, Thermo Fisher Scientific), following the manufacturer’s instructions. Cortisol concentration was measured using a cortisol-competitive human ELISA kit (Cat. No. EIAHCOR, Thermo Fisher Scientific). Catecholamine levels were determined using epinephrine (Cat. No. KA1882), norepinephrine (Cat. No. KA1891), and dopamine (Cat. No. KA3838) ELISA kits (Abnova, Taipei, Taiwan) according to the manufacturer’s protocols. All enzyme-linked immunosorbent assay (ELISA) measurements were performed using a Varioskan LUX multimode microplate reader (Thermo Fisher Scientific).

### 2.5 High-throughput RNA sequencing

Small RNA sequencing libraries were constructed using the NEBNext Multiplex High-Throughput RNA Library Prep Kit (New England Biolabs, Ipswich, MA, United States) according to the manufacturer’s instructions. The library preparation workflow included adapter ligation, reverse transcription, and PCR amplification to optimize the quality and yield of the small RNA libraries. After construction, the libraries were subjected to rigorous quantification and quality validation to ensure their readiness for sequencing. High-throughput sequencing was performed on the NextSeq 500 platform (Illumina, San Diego, CA, United States) to generate extensive RNA sequencing data. The high-resolution data obtained facilitated an in-depth analysis of miRNA expression dynamics and their potential regulatory roles.

### 2.6 Screening for differentially expressed miRNAs

To identify the differentially expressed miRNAs, miRNA expression profiling was performed using data obtained from patients with GI cancer who underwent oxaliplatin-based therapy. For differential expression analysis, this study utilized the edgeR Bioconductor package, an R-based statistical method designed to identify miRNAs with significant dysregulation. ExDEGA software (eBiogen, Seoul, Korea) was used to detect significant changes in miRNA expression levels. miRNA expression profiles were visualized using a volcano plot generated using GraphPad Prism software. A fold-change threshold of >2 and a statistical significance level of *P* < 0.05 were used to identify differentially expressed miRNAs. The adjusted P-values were adjusted for multiple comparisons using the Benjamini-Hochberg correction method. This analytical approach enables the detection of miRNAs with altered expression patterns in response to oxaliplatin treatment. Receiver operating characteristic (ROC) curve analysis was conducted to assess the diagnostic potential of the differentially expressed miRNAs as biomarkers. ROC analysis was used to determine the sensitivity and specificity of each miRNA in patients with GI cancer who underwent oxaliplatin treatment. The statistical significance of differential miRNA expression, ROC curve analysis, and Spearman’s rank-order correlation coefficients between differentially expressed miRNAs and NRS scores were assessed using GraphPad Prism software.

### 2.7 Target prediction of miRNAs

The candidate gene targets of the miRNAs under investigation were predicted using three established computational platforms: miRDB (https://mirdb.org/, accessed on 07 January 2026), miRWalk (http://mirwalk.umm.uni-heidelberg.de/, accessed on 07 January 2026), and TargetScan (https://www.targetscan.org/vert_80/, accessed on 08 January 2026). To improve the accuracy and reliability of target predictions, a consensus approach was adopted to identify overlapping genes through Venn diagram-based comparative analysis (Jia et al., 2021). Gene ontology (GO) enrichment analysis was performed to systematically classify the biological roles and functional relevance of overlapping target genes (Thomas et al., 2007).

### 2.8 Target genes enrichment

To investigate the functional roles of genes associated with the candidate miRNAs, GO enrichment analysis was performed using the DAVID Bioinformatics Resource (https://david.ncifcrf.gov/, accessed on 15 January 2026). This analysis was designed to identify the significantly enriched biological processes, molecular functions, and cellular components related to the target gene set (Ashburner et al., 2000). Pathway enrichment analysis was performed using the Kyoto Encyclopedia of Genes and Genomes (KEGG) database, which was integrated with the Cytoscape software 3.9.1 for network visualization and interpretation. Statistical significance was determined using a two-tailed hypergeometric test with adjustments for multiple comparisons made using the Bonferroni step-down correction. A stringent significance threshold of *P* < 0.001 and a kappa score cutoff of ≥0.4 were applied to ensure the robust and reliable identification of enriched pathways and functional categories.

### 2.9 Protein-protein interactions

To explore the functional relationships among the proteins, enrichment analysis of protein–protein interactions (PPIs) was performed using the STRING database (https://string-db.org/, accessed on 29 January 2024). The STRING platform was used to systematically identify and assess potential interaction networks among target proteins (Huang, 2023). To identify the key genes linked to differentially expressed miRNAs, a PPI network was constructed and subjected to topological analysis using the Maximal Clique Centrality (MCC) method (Alruily et al., 2025).

### 2.10 Statistical analysis

Statistical analyses were performed using IBM SPSS version 31.0 (IBM SPSS Inc., New York, United States) and GraphPad Prism version 6.0 (GraphPad Software, La Jolla, CA, United States) software. In all analyses, a *P*-value of <0.05 was considered statistically significant.

## 3 Results

### 3.1 Study group characteristics

The patients included in this study were diagnosed with stomach, colon, or rectum cancer. Patients were stratified into two groups based on their NRS scores (Supplementary Table S2). Group 1 consisted of 10 patients with no or mild pain, whereas Group 2 included 12 patients who experienced moderate or severe pain. The mean age of Groups 1 and 2 was 60.0 and 61.5 years, respectively. Both groups included males and females. No significant differences in routine blood test parameters were observed between the two groups. Group 2 exhibited a greater number of oxaliplatin treatment cycles than Group 1. Given the highly cumulative nature of oxaliplatin-induced neurotoxicity, the incidence and severity of these symptoms are expected to increase with increasing cumulative drug exposure (Cheng et al., 2023). GI cancer staging was performed according to the American Joint Committee on Cancer (AJCC) classification system, and each diagnosed case was assigned a corresponding stage. The staging distribution and routine blood test parameters are summarized in Supplementary Table S3. Plasma norepinephrine and dopamine levels decreased, and NGF and cortisol levels increased with higher NRS pain scores, highlighting the need for further investigation into the functional role of dopamine in neuropathic pain (Table 1).

**TABLE 1:** Individual plasma neuroendocrine marker values by patient with gastrointestinal cancer who underwent oxaliplatin treatment.

Patient No.	NRS category	NGF (pg/mL)	Epinephrine (pg/mL)	Norepinephrine (pg/mL)	Dopamine (pg/mL)	Cortisol (ng/mL)
1	No pain or mild	202	255	85	73	70
2	No pain or mild	996	153	52	81	75
3	No pain or mild	768	231	87	83	36
4	No pain or mild	1,220	101	34	34	101
5	No pain or mild	1,220	101	34	34	114
6	No pain or mild	352	230	86	106	101
7	No pain or mild	1,268	234	86	82	114
8	No pain or mild	812	116	52	101	90
9	No pain or mild	812	255	85	120	135
10	No pain or mild	352	101	34	26	101
11	Moderate or severe	1,100	59	26	18	90
12	Moderate or severe	1,432	39	16	2	101
13	Moderate or severe	812	20	17	7	110
14	Moderate or severe	1,620	231	87	83	158
15	Moderate or severe	1,164	101	34	43	114
16	Moderate or severe	996	124	48	57	157
17	Moderate or severe	1,620	231	87	106	129
18	Moderate or severe	352	59	26	18	157
19	Moderate or severe	1,268	213	16	12	143
20	Moderate or severe	1,424	113	38	51	145
21	Moderate or severe	1,108	20	17	11	129
22	Moderate or severe	996	213	16	20	152

NGF: nerve growth factor, NRS, numeric rating scale.

### 3.2 Differential miRNA expression in patients with oxaliplatin-treated gastrointestinal cancer

**FIGURE 1:**
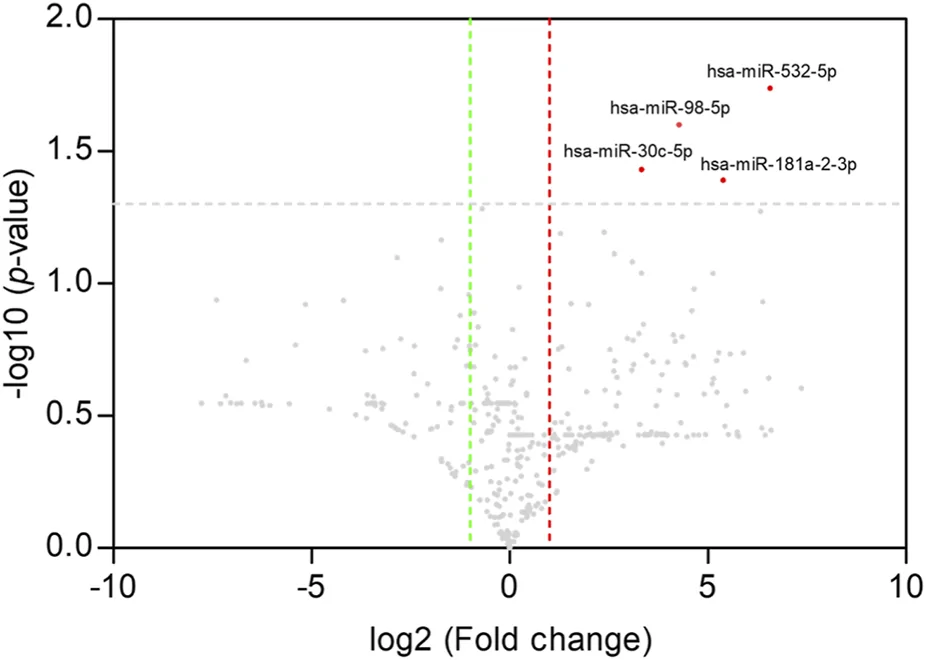
miRNAs exhibiting differential expression in patients with gastrointestinal cancer who underwent oxaliplatin treatment. Red dots indicate miRNAs with increased expression levels, while gray dots represent miRNAs showing no significant changes in expression.

**FIGURE 2:**
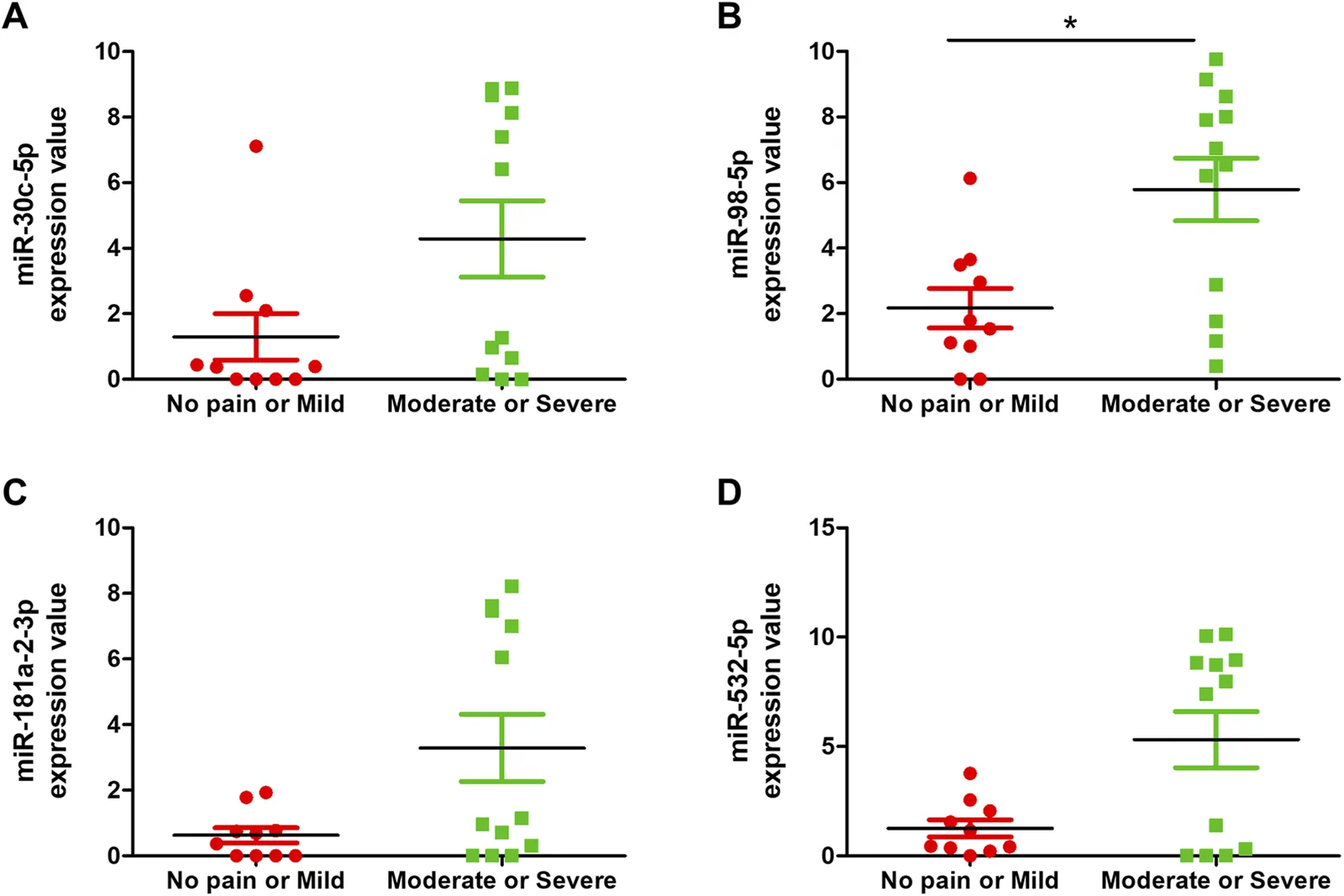
miRNA expression levels in oxaliplatin-treated gastrointestinal cancer patients. **(A)** miR-30c-5p. **(B)** miR-98-5p. **(C)** miR-181a-2-3p. **(D)** miR-532-5p. *P < 0.05.

**FIGURE 3:**
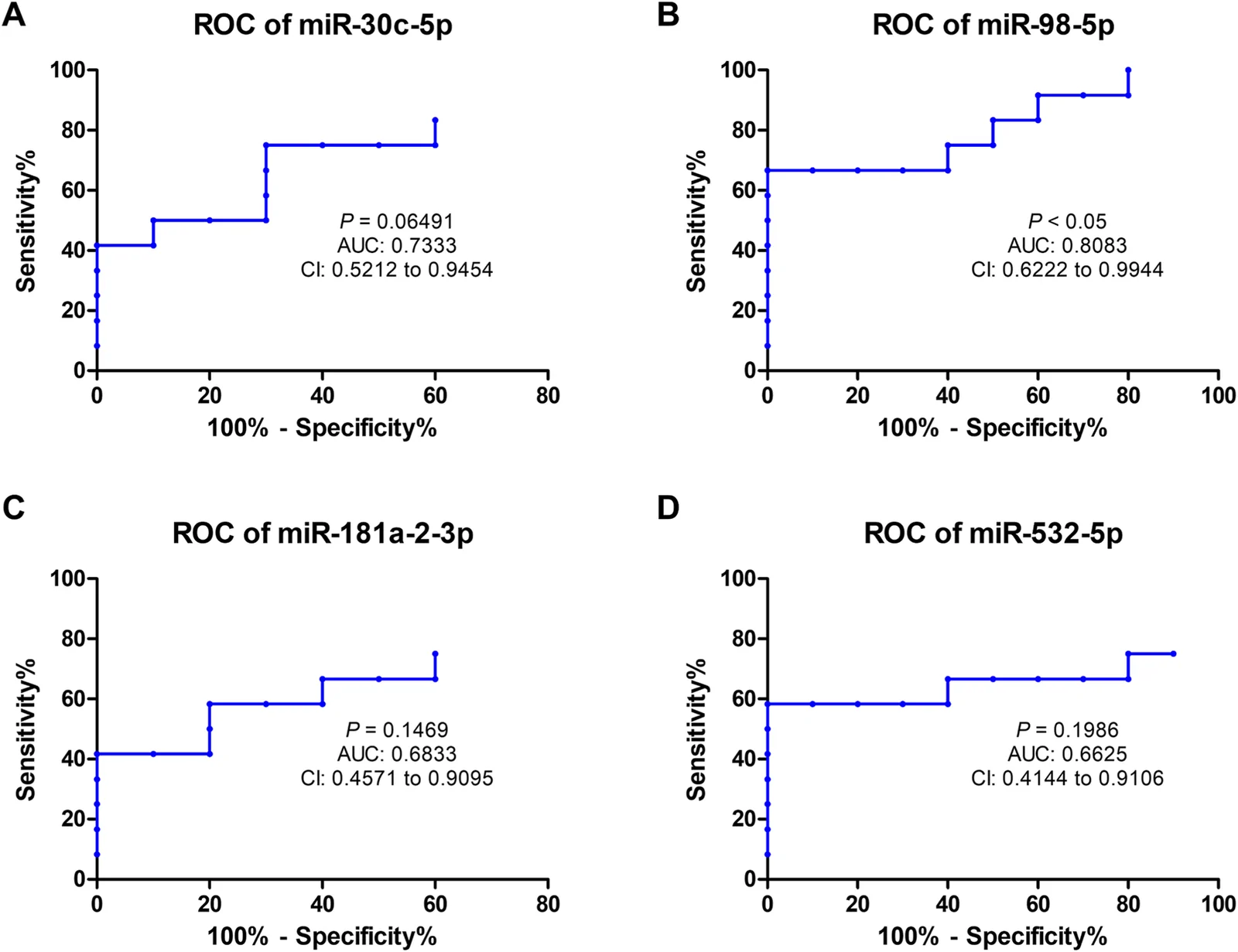
Diagnostic potential of four miRNAs as biomarkers for oxaliplatin-induced peripheral neuropathy. **(A)** miR-30c-5p. **(B)** miR-98-5p. **(C)** miR-181a-2-3p. **(D)** miR-532-5p. *P* < 0.05 was considered significant.

High-throughput RNA sequencing was used to analyze the plasma samples obtained from patients with GI cancer treated with oxaliplatin. Volcano plot analysis revealed four differentially expressed miRNAs, hsa-miR-30c-5p (p-value = 0.0371, adjusted p-value = 0.5186), hsa-miR-98-5p (p-value = 0.0250, adjusted p-value = 0.5186), hsa-miR-181a-2-3p (p-value = 0.0406, adjusted p-value = 0.5186), and hsa-miR-532-5p (p-value = 0.0182, adjusted p-value = 0.5186), which were significantly upregulated (Figure 1). Among these candidates, hsa-miR-98-5p was the only miRNA to reach statistical significance (Figures 2A–D). ROC analysis showed that hsa-miR-98-5p was the most promising candidate, exhibiting superior potential as a stratification biomarker. The area under the curve (AUC) for hsa-miR-98-5p exceeded 0.8 (Figures 3A–D). An AUC value between 0.8 and 0.9 is generally considered excellent (Mandrekar, 2010).

### 3.3 Correlation between miR-98-5p expression and NRS score

To evaluate the potential relationship between miR-98-5p expression and the severity of neuropathic pain, Spearman rank correlation analysis was performed using data from patients with GI cancer who underwent oxaliplatin treatment. The analysis did not reveal a statistically significant correlation between plasma miR-98-5p levels and NRS scores, although a positive trend was observed (r = 0.4031, *P* = 0.0629) (Supplementary Figure S1).

### 3.4 Association of plasma miR-98-5p expression with NGF, catecholamines, and cortisol levels

To investigate the biological significance of plasma miR-98-5p, its correlation with stress- and pain-related mediators was examined using Spearman correlation analysis. Plasma miR-98-5p expression levels showed a significant positive correlation with cortisol (r = 0.546, P = 0.009), suggesting an association between miR-98-5p expression and physiological stress response. A positive but non-significant trend was observed between miR-98-5p and NGF (r = 0.387, P = 0.075). No significant correlations were found between miR-98-5p expression and epinephrine (r = −0.095, P = 0.675), norepinephrine (r = −0.111, P = 0.624), or dopamine (r = −0.022, P = 0.921) (Supplementary Figure S2). These findings indicate that among the mediators examined, cortisol demonstrated the strongest association with plasma miR-98-5p expression, supporting its potential role as a candidate biomarker linked to the stress-related component of pain severity.

### 3.5 Target prediction and enrichment analysis of hsa-miR-98-5p

**FIGURE 4:**
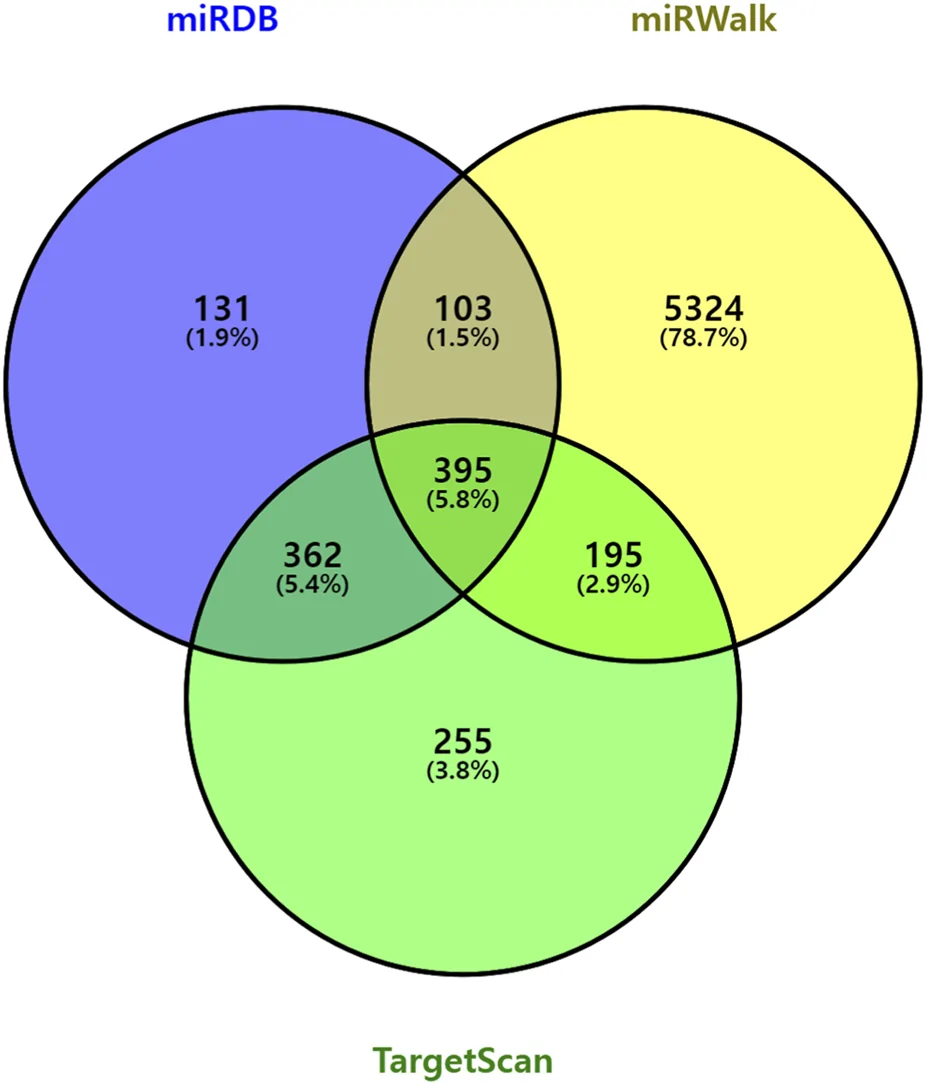
Overlapping target genes of hsa-miR-98-5p. A Venn diagram was constructed to display the overlapping genes identified from the miRDB, TargetScan, and miRWalk databases.

**FIGURE 5:**
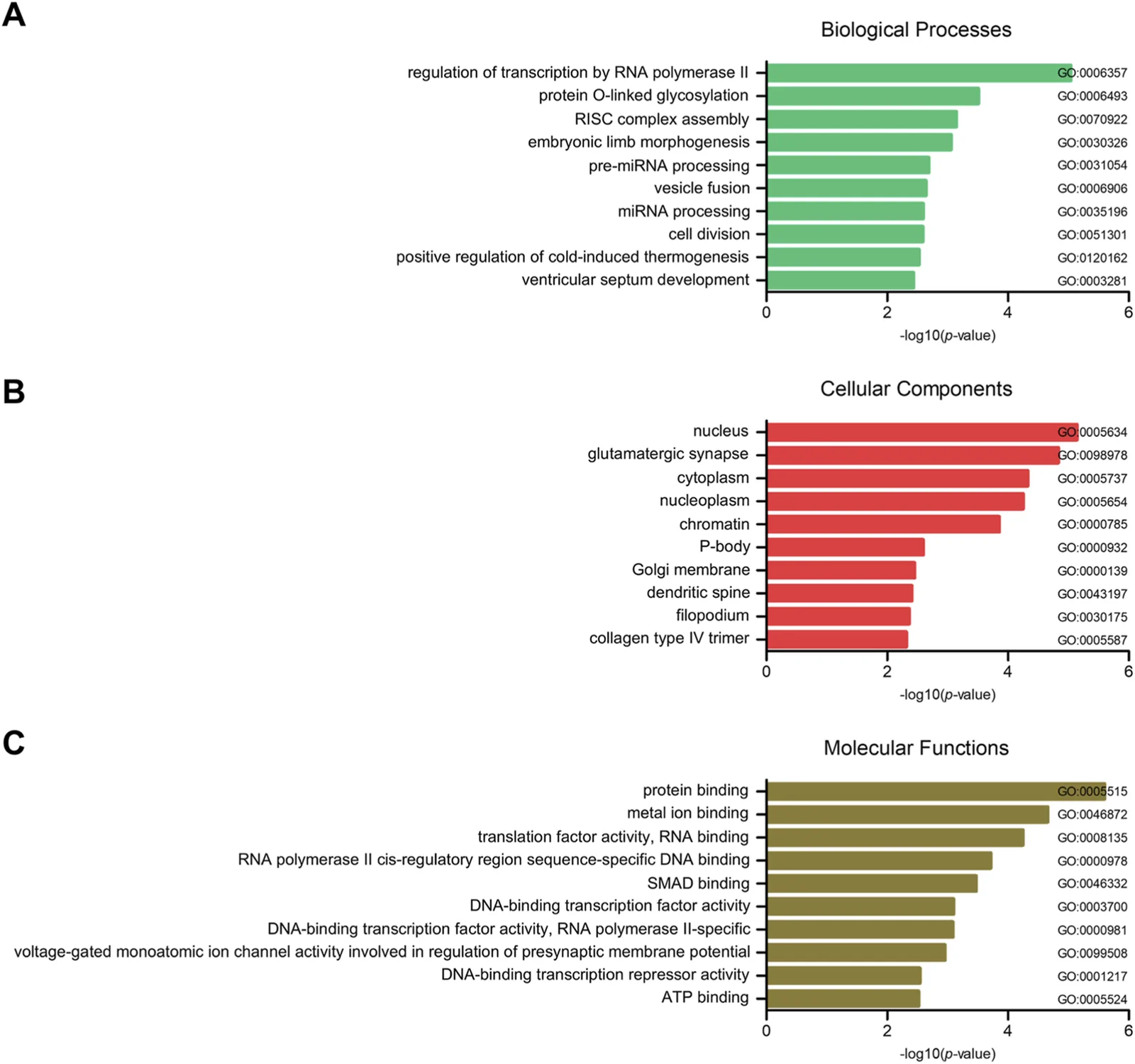
Gene ontology functional annotations of predicted target genes into three distinct categories. **(A)** Biological processes. **(B)** Cellular component. **(C)** Molecular functions.

**FIGURE 6:**
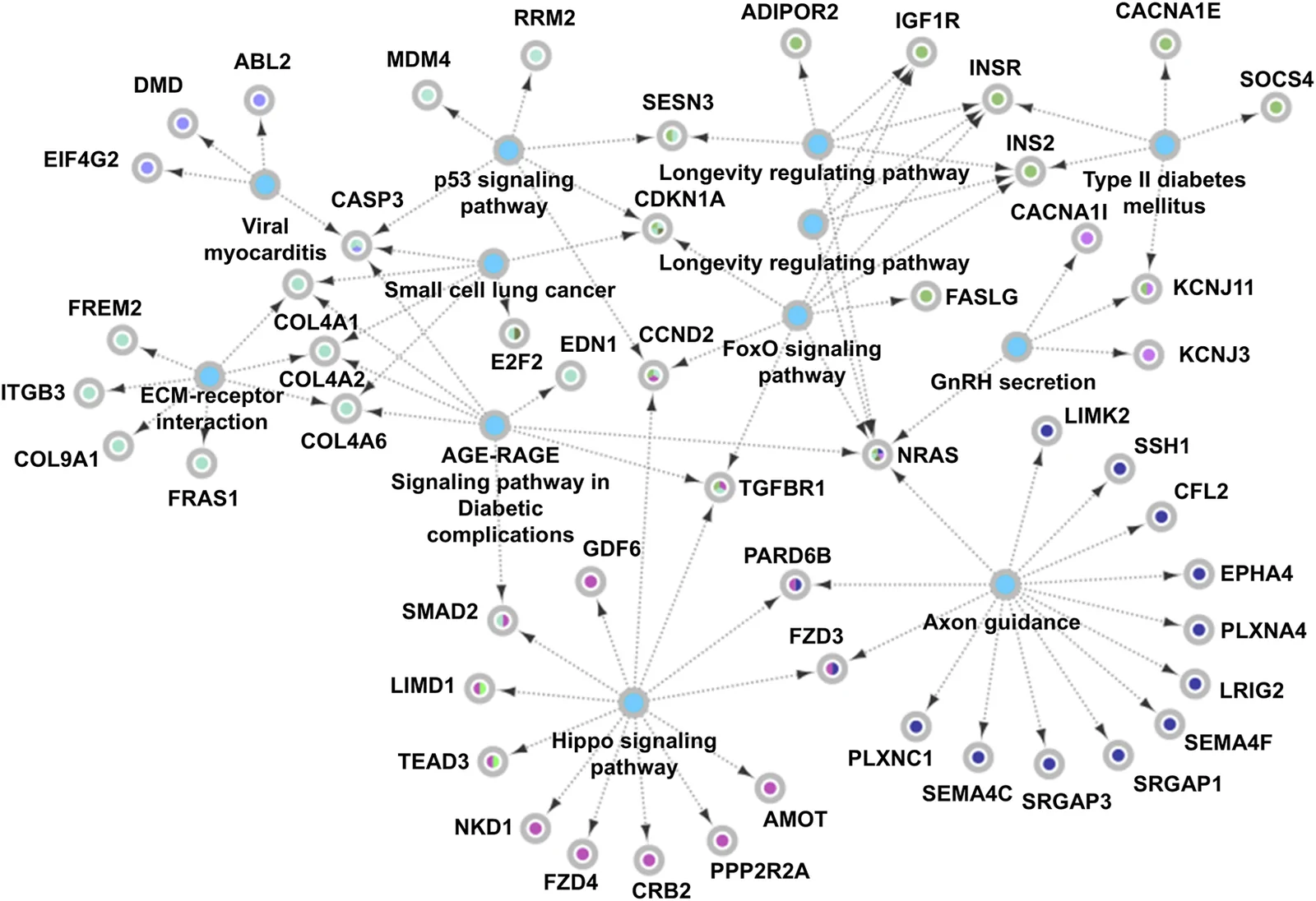
KEGG pathway network analysis of potential target genes of hsa-miR-98-5p.

In total, 395 genes were identified as computationally predicted overlapping targets of hsa-miR-98-5p (Figure 4). These predictions represent *in silico* analyses and have not been experimentally confirmed in OIPN patients. These findings are based on computational prediction and require experimental validation before causal relationships can be established. GO enrichment analysis identified significant enrichment of these genes in key biological processes, including transcription regulation by RNA polymerase II (GO:0006357), protein O-linked glycosylation (GO:0006493), and RISC complex assembly (GO:0070922) (Figure 5A). Cellular component analysis revealed prominent associations with the nucleus (GO:0005634), glutamatergic synapses (GO:0098978), and cytoplasm (GO:0005737) (Figure 5B). At the molecular level, the enriched functions included interactions such as protein binding (GO:0005515), metal ion binding (GO:0046872), translation factor activity, and RNA binding (GO:0008135) (Figure 5C). Pathway enrichment analysis of the target genes revealed significant involvement of the p53 signaling pathway (KEGG:04,115), which is associated with cellular stress responses and apoptosis regulation. Axon guidance (KEGG:04,360) was identified as a key pathway, highlighting its potential role in neuronal development and repair. The Hippo signaling pathway (KEGG:04,390) was also significantly enriched, suggesting its contribution to the control of cellular proliferation (Figure 6; Supplementary Table S4). These findings highlight the diverse functional pathways through which target genes influence biological processes.

### 3.6 Protein–protein interaction network analysis

**FIGURE 7:**
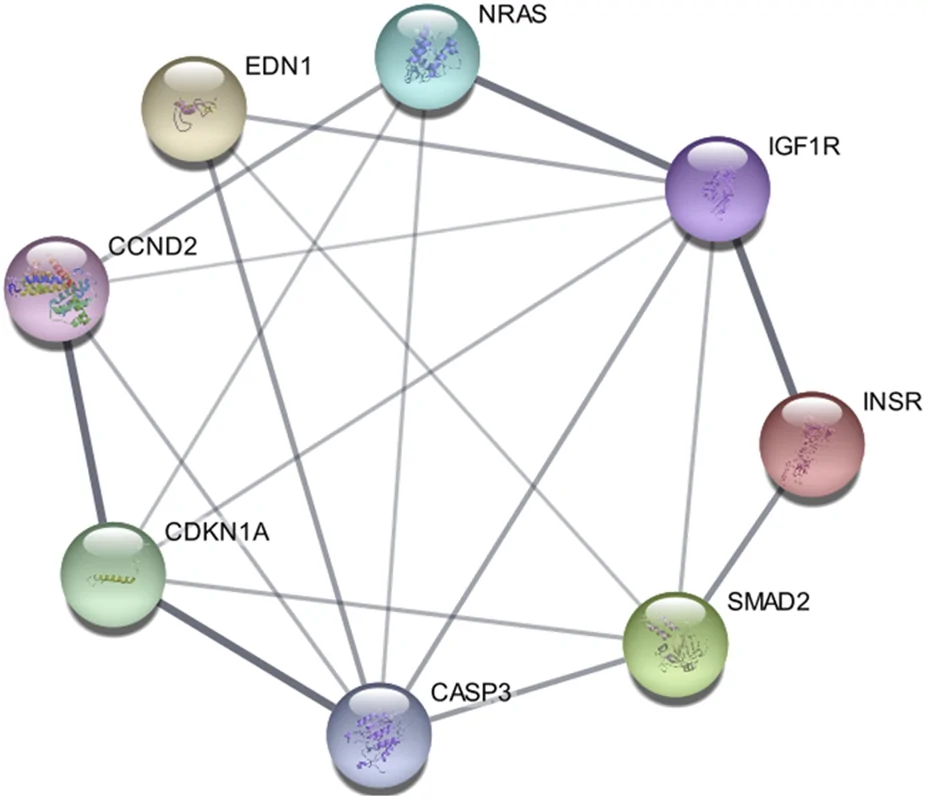
Identification of hub genes using protein-protein interaction network. The protein-protein interaction network was analyzed using the MCC algorithm to identify hub genes.

PPI network analysis identified eight highly interconnected hub genes: NRAS, IGF1R, INSR, SMAD2, CASP3, CDKN1A, CCND2, and EDN1 (Figure 7). The eight hub genes identified through the PPI network using the MCC method were derived solely from network topology analysis. In models of chemotherapy-induced and nerve injury-induced neuropathy, insulin-like growth factor 1 receptor (IGF1) signaling through its receptor has been shown to affect pain-like behaviors, with IGF1R predominantly expressed on neurons (Chen et al., 2021; Jiang et al., 2024). Endothelin-1 (EDN1) has an established role in pain modulation, with involvement documented across multiple pain conditions including inflammatory pain, cancer-related pain, neuropathic pain and diabetic neuropathy, suggesting that EDN1 signaling may represent a potential therapeutic target for the relief of various forms of chronic pain (Smith et al., 2014). However, these genes were not subjected to further experimental validation, such as expression-level analysis in a relevant biological context, their identification should be considered hypothesis-generating rather than confirmatory. Further exploration of these hub genes may reveal novel severity stratification biomarkers or therapeutic targets associated with the hsa-miR-98-5p-related pathways.

## 4 Discussion

Oxaliplatin-induced peripheral neurotoxicity (OIPN) significantly compromises the quality of life and treatment outcomes of patients, highlighting the need for innovative approaches for its prevention and management. OIPN manifests in two distinct clinical types. Acute OIPN is driven by ion channel dysfunction, whereas chronic OIPN is characterized by mitochondrial dysfunction, oxidative stress, and neuroinflammation (Wang et al., 2018; Sabina et al., 2022). Considering the established neurotoxic side effects of oxaliplatin, a deeper understanding of the molecular alterations induced by this chemotherapeutic agent is required. This study provides critical insights into the molecular pathways underlying OIPN in patients with gastrointestinal (GI) cancer. It emphasizes the role of differentially expressed microRNAs (miRNAs) as promising candidates for biomarker of pain severity in OIPN. Through high-throughput RNA sequencing analysis, we identified four differentially expressed miRNAs, namely, hsa-miR-30c-5p, hsa-miR-98-5p, hsa-miR-181a-2-3p, and hsa-miR-532-5p, in the plasma of GI cancer patients who underwent oxaliplatin-based chemotherapy. Although none of the candidates achieved statistical significance after FDR correction at the screening stage, this may reflect the limited sample size rather than a lack of biological relevance. Notably, hsa-miR-98-5p showed a consistent association with pain severity, supporting its potential as a candidate biomarker warranting validation in an independent, adequately powered cohort. hsa-miR-98-5p was significantly upregulated in patients with moderate-to-severe neuropathic pain and demonstrated diagnostic potential, as evidenced by an area under the receiver operating characteristic curve exceeding 0.8. The 95% CI of the AUC for hsa-miR-98-5p spans from 0.6222 to 0.9944, reflecting substantial statistical uncertainty attributable to the small sample size (n = 22). These findings should be considered exploratory and hypothesis-generating rather than definitive evidence of clinical utility. hsa-miR-98-5p, a member of the let-7 miRNA family, has been previously shown to promote cisplatin resistance in epithelial ovarian cancer cells (Yang et al., 2021). The potential use of hsa-miR-98-5p as a candidate biomarker of pain severity in OIPN was validated in patients with chronic neuropathic or nociceptive pain (Jiang et al., 2025). The possible involvement of hsa-miR-98-5p in the pathogenesis of OIPN has been suggested, along with its utility as a biomarker for monitoring neurotoxicity.

GO enrichment analysis revealed that target genes were significantly associated with several key biological processes, cellular components, and molecular functions. These processes are crucial in cellular signaling and development and may play roles in neuronal function and neurotoxicity. The association between Kyoto Encyclopedia of Genes and Genomes (KEGG) pathways and neuropathy has been extensively studied, revealing critical molecular mechanisms underlying nerve damage and dysfunction. Key pathways such as axon guidance, the p53 signaling pathway, and the Hippo signaling pathway have been implicated in the pathogenesis of neuropathy, providing potential therapeutic targets for mitigating neurotoxic effects. Axonal guidance signaling is a critical pathway responsible for directing axons to their synaptic targets and may contribute to the pathogenesis of neurotoxicity in patients who have received chemotherapy (Liu et al., 2023). Alterations in the expression or function of axon-guidance proteins may lead to pathological changes in neural circuits, causing neurological disorders (Van Battum et al., 2015). p53 plays a critical role in the regulation of multiple signaling pathways that are essential for maintaining the physiological homeostasis of nerve cells (Li et al., 2023). The Hippo signaling pathway plays a crucial role in the regulation of biological processes, including the development and transformation of the nervous system (Wei et al., 2023). These findings offer a comprehensive understanding of the development of OIPN and highlight several critical pathways and target genes. The PPI network analysis identified eight interconnected hub genes, among which IGF1R and EDN1 are of particular interest given their previously reported roles in pain signaling, including neuropathic and chemotherapy-induced pain models. These findings suggest that hsa-miR-98-5p may influence OIPN severity in part through IGF1R- or EDN1-related pathways.

Peripheral neuropathy-induced alterations in the autonomic system may contribute to the chronic maladaptation to persistent pain (Sagheddu et al., 2015). Sympathetic dysregulation modulates pain processing by altering the propagation of nociceptive signals, suggesting a neuromodulatory role for dopamine and norepinephrine in the maintenance of chronic pain (Jarcho et al., 2012). Given this potential role, measurement of plasma catecholamine levels could support the diagnosis and development of targeted therapies for neurological disorders (Dias et al., 2020). Norepinephrine is primarily released from sympathetic nerve terminals, where it mediates physiological responses in organs and tissues (Yuan et al., 2025). One study has demonstrated that norepinephrine plays a critical role in the suppression of neuropathic pain (Obata, 2017). Dopamine modulates synaptic transmission in dorsal root ganglion neurons, thereby influencing pain signaling (Wang et al., 2021). Dopamine D1 and D2 receptor complexes drive chronic neuropathic pain through Gαq-mediated activation of PKCγ-CaMKII-MAPK-CREB signaling cascades (Bao et al., 2021). Dopamine enhances the noradrenergic effects to inhibit neuropathic pain (Obata, 2017). NGF inhibition attenuates neuropathic pain through TAK1-dependent suppression of MAPK and p65 signaling (Dai et al., 2020). Excessive cortisol production disrupts the HPA axis regulation and induces neuroinflammation in the central nervous system, thereby promoting the progression of neurodegenerative diseases (Knezevic et al., 2023).

**FIGURE 8:**
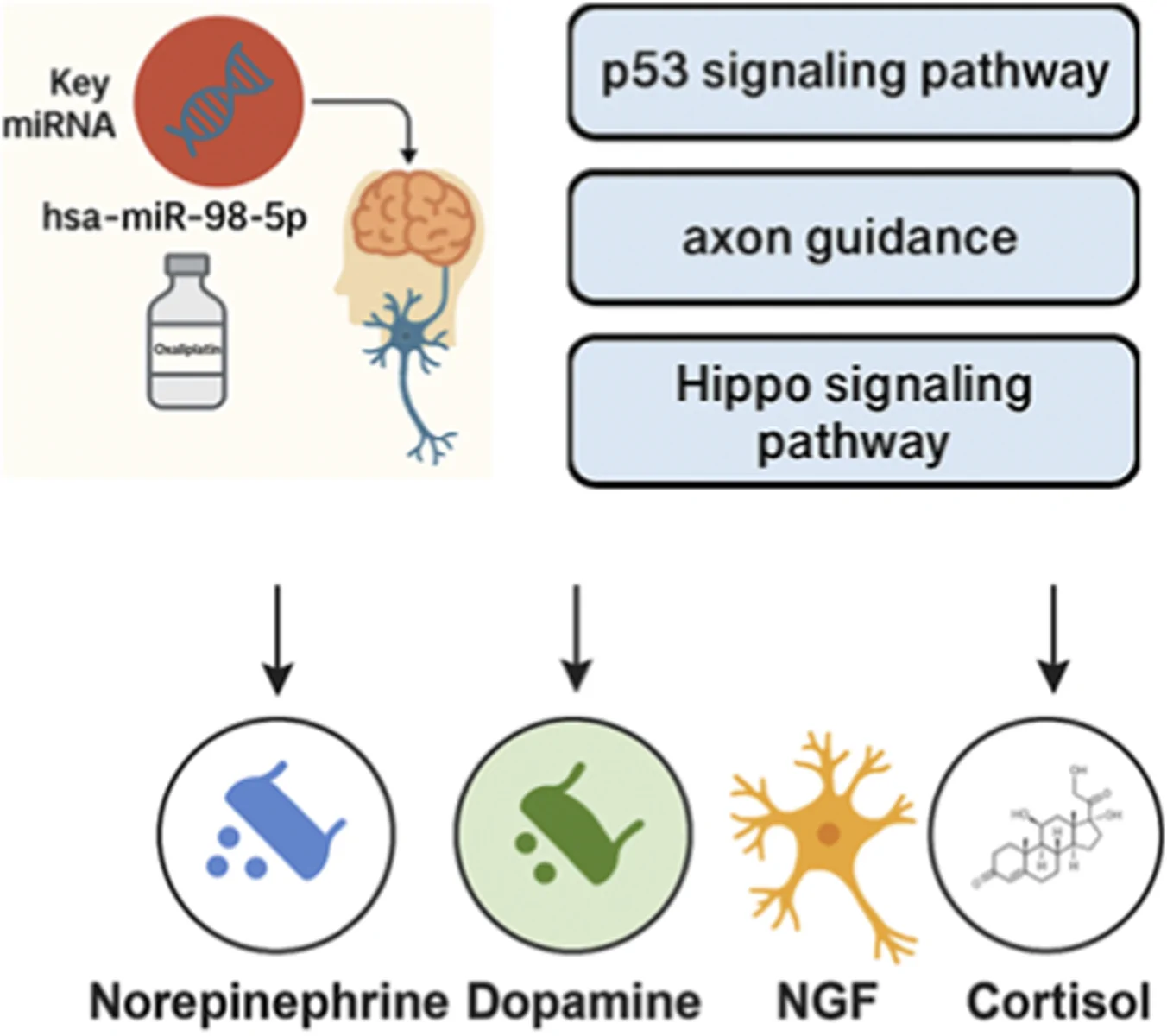
An overview of oxaliplatin-induced pain sensitivity.

In the present cohort, patients with moderate or severe pain exhibited lower plasma concentrations of norepinephrine and dopamine, along with elevated NGF and cortisol levels, compared with those with no or mild pain. Plasma miR-98-5p expression was significantly correlated with cortisol. Because this study employed a cross-sectional design, the directionality of these associations cannot be determined from the present data. It is possible that alterations in NGF and catecholamine levels contribute to the development or maintenance of neuropathic pain, as proposed in the mechanistic literature cited above. However, it is equally possible that these neurochemical changes represent a consequence of pain itself, for example, through HPA-axis activation and altered sympathetic tone secondary to chronic nociceptive input, rather than its cause. The mechanistic interpretations offered here are therefore hypotheses drawn from external experimental literature rather than conclusions supported by the present cross-sectional data. Regardless of directionality, NGF and catecholamines may still hold value as correlative biomarkers of neuropathic severity, with their potential utility as therapeutic targets requiring confirmation in studies capable of establishing temporal or causal relationships. Collectively, these findings suggest that miR-98-5p may contribute to peripheral sensitization in part by interacting with these neurochemical mediators, and that a composite biomarker panel incorporating miR-98–5p, catecholamines, NGF, and cortisol may hold value for both the early detection of OIPN and the monitoring of pain progression (Figure 8).

Several limitations warrant consideration. All miRNA-target gene interactions reported here are computationally predicted and have not been experimentally validated in OIPN patient samples or neuronal cell models. Future studies employing luciferase reporter assays or paired miRNA-mRNA expression profiling from the same patients are needed to confirm these interactions and to delineate the direct regulatory functions of hsa-miR-98-5p within neuropathic pain pathways. The sample size was also relatively small, and the findings require validation in a larger, independent cohort with functional studies confirming the regulatory roles of hsa-miR-98-5p and its target genes in neuronal injury models.

To the best of our knowledge, this is the first study to integrate NRS-based pain severity with plasma miRNA expression and NGF and catecholamine profiling in patients with GI cancer treated with oxaliplatin. This integrative approach offers new insights into the molecular mechanisms underlying OIPN and may support the development of more precise diagnostic and therapeutic strategies.

## Data Availability

The datasets generated and/or analyzed during the current study are available in the following public repositories: the small RNA sequencing data have been deposited in the NCBI Sequence Read Archive (SRA) under BioProject accession no. PRJNA1425790, and the corresponding miRNA expression profile is available in the NCBI Gene Expression Omnibus (GEO) repository under accession number GSE319960 (https://www.ncbi.nlm.nih.gov/geo/query/acc.cgi?acc=GSE319960).
