# Supplementary material for: Plasma miR-98-5p as a candidate biomarker of pain severity in oxaliplatin-induced peripheral neuropathy related to NGF and catecholamine dysregulation
Source: Front Mol Biosci. 2026 Sep 8;13:1884789. doi: 10.3389/fmolb.2026.1884789 (PMC13593450; doi:10.3389/fmolb.2026.1884789)
Supplement: Supplementary file 1 [file DataSheet1.docx]

**Supplemental Information**

**Plasma miR-98-5p as a candidate biomarker of pain severity in oxaliplatin-induced peripheral neuropathy related to NGF and catecholamine dysregulation**

**Yeongdon Ju, Go-Eun Choi and Aelee Jang**

**Supplementary Table S1. Sample-level relationship among the three related studies**

| Study | Patient population | Overlap with present study | microRNA profiling of plasma samples |
| --- | --- | --- | --- |
|  |  |  |  |
| Ju et al.  (2022) | XELOX-treated gastric cancer | 4 patients shared with present cohort | Original profiling performed in Ju et al. (2022) |
| Ju et al.  (2024) | FOLFOX-treated colorectal cancer | 12 patients shared with present cohort | Original profiling performed in Ju et al. (2024) |
| Present study | Oxaliplatin-treated  gastrointestinal cancer | 6 newly enrolled patients | New, independent miRNA profiling performed for all 22 patients in the present study |

**Supplementary Table S2. Numerical rating scale (NRS) ratings of patients with gastrointestinal cancer who underwent oxaliplatin treatment**

| Patients | Age (years) | Sex | Numerical Rating Scale | | Oxaliplatin | Dysfunction | | |
| --- | --- | --- | --- | --- | --- | --- | --- | --- |
|  |  |  | Group 1 | Group 2 |  | Motor | Sensory | Autonomic |
| 1 | 44 | Female | 0 |  | 2 cycles | Normal | Normal | Normal |
| 2 | 32 | Male | 1 |  | 2 cycles | Normal | + | Normal |
| 3 | 66 | Male | 3 |  | 4 cycles | Normal | + | Normal |
| 4 | 62 | Female | 0 |  | 2 cycles | Normal | Normal | Normal |
| 5 | 68 | Female | 2 |  | 3 cycles | Normal | + | Normal |
| 6 | 56 | Female | 2 |  | 3 cycles | Normal | + | Normal |
| 7 | 58 | Male | 0 |  | 2 cycles | Normal | Normal | Normal |
| 8 | 59 | Female | 0 |  | 2 cycles | Normal | Normal | Normal |
| 9 | 61 | Male | 3 |  | 3 cycles | Normal | + | Normal |
| 10 | 58 | Male | 3 |  | 6 cycles | Normal | + | Normal |
| 11 | 32 | Male |  | 5 | 4 cycles | + | + | Normal |
| 12 | 32 | Male |  | 6 | 5 cycles | + | + | + |
| 13 | 66 | Male |  | 6 | 7 cycles | + | + | + |
| 14 | 64 | Male |  | 5 | 3 cycles | + | + | Normal |
| 15 | 71 | Male |  | 4 | 3 cycles | + | + | Normal |
| 16 | 62 | Female |  | 7 | 7 cycles | + | + | + |
| 17 | 68 | Female |  | 5 | 7 cycles | + | + | + |
| 18 | 56 | Female |  | 6 | 8 cycles | + | + | + |
| 19 | 64 | Male |  | 8 | 8 cycles | + | + | + |
| 20 | 71 | Male |  | 6 | 7 cycles | + | + | + |
| 21 | 59 | Female |  | 6 | 6 cycles | + | + | Normal |
| 22 | 61 | Male |  | 8 | 8 cycles | + | + | + |

Gruoup 1: No pain or mild, Group 2: Moderate or severe

**Supplementary Table S3. Clinical characteristics of patients with gastrointestinal cancer who underwent oxaliplatin treatment**

| Variables:  Mean ± SD | Group 1 (*n* = 10)  (No pain or Mild) | Group 2 (*n* = 12)  (Moderate or Severe) | *P* value |
| --- | --- | --- | --- |
| Age (years) |  |  | 0.647 |
| Median | 60.0 | 61.5 |  |
| Range | 32–68 | 32–71 |  |
| Sex |  |  | 0.453 |
| Female | 5 | 4 |  |
| Male | 5 | 8 |  |
| Primary tumor |  |  | 0.533 |
| Stomach | 2 | 2 |  |
|  |  |  |  |
| Colon | 6 | 6 |  |
| Rectum | 2 | 4 |  |
|  |  |  |  |
| Depth of invasion (T) |  |  | 0.207 |
| T2 | 1 | 3 |  |
| T3 | 2 | 4 |  |
| T4 | 2 | 1 |  |
| T4a | 5 | 4 |  |
| Lymph node metastasis (N) |  |  | 0.327 |
| N0 | 5 | 3 |  |
| N1 | 1 | 3 |  |
| N1b | 2 | 2 |  |
| N2b | 1 | 2 |  |
| N3a | 1 | 2 |  |
| TNM stage |  |  | 0.739 |
| I | 1 | 1 |  |
| IIA | 1 | 1 |  |
| IIB | 3 | 1 |  |
| IIIA | 0 | 2 |  |
| IIIB | 2 | 2 |  |
| IIIC | 0 | 2 |  |
| IVA | 1 | 2 |  |
| IVB | 2 | 1 |  |
| Blood test |  |  |  |
| WBC (cells/mL) | 5399.00 ± 2558.85 | 4923.33 ± 2349.20 | 0.655 |
| Hb (g/dL) | 12.18 ± 1.18 | 11.94 ± 1.29 | 0.658 |
| PLT (cells/mL) | 377900.00 ± 580725.49 | 157583.33 ± 74986.61 | 0.206 |
| Neutrophil/Lymphocyte Ratio | 1.76 ± 0.84 | 1.92 ± 1.36 | 0.757 |
| CRP (mg/dL) | 0.18 ± 0.31 | 0.22 ± 0.25 | 0.762 |
| AST (U/L) | 32.00 ± 20.08 | 37.08 ± 14.05 | 0.494 |
| ALT (U/L) | 27.80 ± 23.23 | 26.67 ± 10.50 | 0.881 |
| TB (mg/dL) | 9.91 ± 29.90 | 1.14 ± 1.94 | 0.654 |
| BST (mg/dL) | 106.10 ± 19.31 | 109.17 ± 21.67 | 0.732 |
| Albumin (g/dL) | 4.30 ± 0.19 | 4.22 ± 0.27 | 0.449 |
| Total Protein (g/dL) | 6.83 ± 0.38 | 6.80 ± 0.26 | 0.840 |
| BUN (mg/dL) | 15.11 ± 4.13 | 15.11 ± 3.35 | 0.999 |
| Creatinine (mg/dL) | 0.86 ± 0.28 | 0.89 ± 0.24 | 0.793 |
| LDH (U/L) | 202.20 ± 37.52 | 220.58 ± 39.90 | 0.282 |
| Na (mmol/L) | 139.97 ± 1.24 | 140.25 ± 1.97 | 0.701 |
| K (mmol/L) | 4.51 ± 0.44 | 4.46 ± 0.46 | 0.814 |
| Ca^2^ (mmol/L) | 9.43 ± 0.30 | 9.43 ± 0.40 | 0.972 |

**Supplementary Table S4. KEGG pathway enrichment analysis of target genes in patients with gastrointestinal cancer who underwent oxaliplatin treatment**

| KEGG ID | Term | Associated gene | *P* value |
| --- | --- | --- | --- |
| KEGG:04115 | p53 signaling pathway | CASP3, CCND2, CDKN1A, MDM4, RRM2, SESN3 | 0.00385 |
| KEGG:04360 | Axon guidance | CFL2, EPHA4, FZD3, LIMK2, LRIG2, NRAS, PARD6B, PLXNA4, PLXNC1, SEMA4C, SEMA4F, SRGAP1, SRGAP3, SSH1 | 0.00002 |
| KEGG:04390 | Hippo signaling pathway | AMOT, CCND2, CRB2, FZD3, FZD4, GDF6, LIMD1, NKD1, PARD6B, PPP2R2A, SMAD2, TEAD3, TGFBR1 | 0.00002 |
| KEGG:04929 | GnRH secretion | CACNA1I, KCNJ11, KCNJ3, NRAS | 0.04282 |
| KEGG:05416 | Viral myocarditis | ABL2, CASP3, DMD, EIF4G2 | 0.03499 |
| KEGG:04512 | ECM-receptor interaction | COL4A1, COL4A2, COL4A6, COL9A1, FRAS1, FREM2, ITGB3 | 0.00220 |
| KEGG:04933 | AGE-RAGE signaling pathway in diabetic complications | CASP3, COL4A1, COL4A2, COL4A6, EDN1, NRAS, SMAD2, TGFBR1 | 0.00104 |
| KEGG:05222 | Small cell lung cancer | CASP3, CDKN1A, COL4A1, COL4A2, COL4A6, E2F2 | 0.01172 |
| KEGG:04068 | FoxO signaling pathway | CCND2, CDKN1A, FASLG, IGF1R, INSR, IRS2, NRAS, TGFBR1 | 0.00564 |
| KEGG:04211 | Longevity regulating pathway | ADIPOR2, IGF1R, INSR, IRS2, NRAS, SESN3 | 0.01005 |
| KEGG:04213 | Longevity regulating pathway | IGF1R, INSR, IRS2, NRAS | 0.03879 |
| KEGG:04930 | Type II diabetes mellitus | CACNA1E, INSR, IRS2, KCNJ11, SOCS4 | 0.00245 |

**Supplementary Figure S1. Spearman's rank-order correlation analysis of miRNA-98-5p expression levels and NRS score.**

**Supplementary Figure S2. Correlation analysis between plasma miR-98-5p expression levels and stress- and pain-related mediators.**
